# Evidence that adiponectin receptor 1 activation exacerbates ischemic neuronal death

**DOI:** 10.1186/2040-7378-2-15

**Published:** 2010-08-11

**Authors:** John Thundyil, Sung-Chun Tang, Eitan Okun, Kausik Shah, Vardan T Karamyan, Yu-I Li, Trent M Woodruff, Stephen M Taylor, Dong-Gyu Jo, Mark P Mattson, Thiruma V Arumugam

**Affiliations:** 1 School of Biomedical Sciences, University of Queensland, Brisbane, Queensland 4072, Australia; 2 Department of Neurology and Stroke Center and Department of Pathology, National Taiwan University Hospital and National Taiwan University College of Medicine, Taipei, Taiwan; 3 Laboratory of Neurosciences, National Institute on Aging, Intramural Research Program, Baltimore, MD 21224, USA; 4 Department of Pharmaceutical Sciences, School of Pharmacy, Texas Tech University Health Sciences Center, Amarillo, Texas 79106, USA; 5 College of Pharmacy, Sungkyunkwan University, 300 Cheoncheon-dong, Jangan-gu, Suwon 440-746, Republic of Korea

## Abstract

**Background-:**

Adiponectin is a hormone produced in and released from adipose cells, which has been shown to have anti-diabetic and anti-inflammatory actions in peripheral cells. Two cell surface adiponectin receptors (ADRs) mediate the majority of the known biological actions of adiponectin. Thus far, ADR expression in the brain has been demonstrated in the arcuate and the paraventricular nucleus of hypothalamus, where its activation affects food intake. Recent findings suggest that levels of circulating adiponectin increase after an ischemic stroke, but the role of adiponectin receptor activation in stroke pathogenesis and its functional outcome is unclear.

**Methods-:**

Ischemic stroke was induced in C57BL/6 mice by middle cerebral artery occlusion (MCAO) for 1 h, followed by reperfusion. Primary cortical neuronal cultures were established from individual embryonic neocortex. For glucose deprivation (GD), cultured neurons were incubated in glucose-free Locke's medium for 6, 12 or 24 h. For combined oxygen and glucose deprivation (OGD), neurons were incubated in glucose-free Locke's medium in an oxygen-free chamber with 95% N2/5% CO_2 _atmosphere for either 3, 6, 9, 12 or 24 h. Primary neurons and brain tissues were analysed for Adiponectin and ADRs using reverse transcriptase polymerase chain reaction (RT-PCR), immunoblot and immunochemistry methods.

**Results-:**

Cortical neurons express ADR1 and ADR2, and that the levels of ADR1 are increased in neurons in response to *in vitro *or *in vivo *ischemic conditions. Neurons treated with either globular or trimeric adiponectin exhibited increased vulnerability to oxygen and glucose deprivation which was associated with increased activation of a pro-apoptotic signaling cascade involving p38 mitogen-activated protein kinase (p38MAPK) and AMP-activated protein kinase (AMPK).

**Conclusions-:**

This study reveals a novel pathogenic role for adiponectin and adiponectin receptor activation in ischemic stroke. We show that cortical neurons express ADRs and reveal a pro-apoptotic role for ADR1 activation in neurons, which may render them vulnerable to ischemic death.

## Introduction

Adiponectin is an abundantly expressed adipokine that is released into the circulation and self-associates to form homotrimers. Adiponectin trimers further associate to form hexamers, high molecular weight (HMW) oligomers and a globular fraction, generated by proteolytic cleavage of full-length adiponectin monomers [1,2]. Adiponectin receptor 1 (ADR1) and adiponectin receptor 2 (ADR2) are the major receptors for adiponectin. Both ADRs can be activated by all forms of adiponectin found in the circulation. However, ADR1 has a higher affinity for globular adiponectin (gAd) over the full-length forms, whereas ADR2 has a similar affinity for both isoforms [3]. In addition, HMW oligomers are reported to be a specific ligand for T-cadherin [4]. ADRs were shown to exert actions in the peripheral tissues by activating the AMP-activated protein kinase α (AMPKα) [5], p38 mitogen-activated protein kinase (p38-MAPK) [6] and nuclear factor-kappa B (NFκB) [reviewed in reference [7]]. In the brain, ADRs 1 and 2 are expressed in the arcuate and the paraventricular nuclei of the hypothalamus, where they regulate feeding behaviours [8,9]. However, the functions of adiponectin in other regions of the central nervous system (CNS) are still poorly understood.

The cerebral ischemia that occurs in brain cells affected by a stroke triggers a complex array of molecular and cellular alterations including activation of signaling pathways that may either contribute to neuronal damage or protect neurons. Among the pathways known to be activated in neurons in response to ischemia, are those involving AMPKα and P38-MAPK [10,11]. It was recently reported that levels of circulating adiponectin increase after an ischemic stroke [12]. However, it is not known whether ADRs are activated in neurons in response to ischemic stroke, nor have the consequences of ADR signaling on the clinical outcome of a cerebral ischemic event been established. In the present study we show that both ADR1 and ADR2 are expressed in cerebral cortical neurons, and that activation of ADR1 leads to neuronal cell death under ischemic conditions.

## Materials and methods

### Animals and Stroke Model

Three-month-old C57BL/6 male mice were used for all *in vivo *experiments. All animal experimental procedures performed were reviewed and approved by the University of Queensland Animal Care and Use Committee. Transient focal cerebral ischemia was induced by middle cerebral artery occlusion (MCAO) using the previously described intraluminal filament method [13]. Briefly, mice were anesthetized with isoflurane, a midline incision was made in the neck, and the left external carotid and pterygopalatine arteries were isolated and ligated with 5-0 silk thread. The internal carotid artery (ICA) was occluded at the peripheral site of the bifurcation of the ICA and the pterygopalatine artery with a small clip, and the common carotid artery (CCA) was ligated with 5-0 silk thread. The external carotid artery (ECA) was cut, and a 6-0 nylon monofilament with a tip that was blunted (0.2-0.22 mm) with a coagulator was inserted into the ECA. After the clip at the ICA was removed, the nylon thread was advanced into the middle cerebral artery (MCA) until light resistance was felt. The nylon thread and the CCA ligature were removed after 1 h of occlusion to initiate reperfusion. In the sham operated group, these arteries were surgically exposed but not disturbed. At different time points during the reperfusion period, mice were euthanized and brains were immediately removed and processed for immunoblot and immunohistochemical analysis.

### Patient tissue collection

The case is a 39-year-old man who had an acute brainstem stroke. He died on the ninth day after the incident; due to massive infarcts and obstructive hydrocephalus. Acute basilar artery occlusion related to atherosclerosis and associated thrombi were suggested as the possible causes of death on autopsy [14].

### Neuronal cultures

Cortical tissues dissected from C57BL/6 mouse embryos at the E15 developmental stage were incubated for 15 min in a solution of 2 mg/ml trypsin in-Ca^2+/^Mg^2+^-free Hank's balanced salt solution (HBSS) (Invitrogen, USA) buffered with 10 mmol/L HEPES. Tissues were then dissociated and cells were plated in 60 or 100-mm diameter plastic dishes or 24-well plates and maintained at 37°C in Neurobasal medium containing B-27 supplements (Invitrogen, Carlsbad, CA, USA), 2 mmol/L L-glutamine, 0.001% gentamycin sulfate and 1 mmol/L HEPES (pH 7.2). Experiments were performed in 7 to 9-day-old cultures. Approximately 95% of the cells in such cultures were neurons and the remaining cells were astrocytes.

### RT-PCR analysis

PCR primers were designed using Primer3 software and synthesized by Integrated DNA Technologies, Inc. (Coralville, IA, USA). Total RNA from cultured neurons was extracted using Trizol reagent (Sigma, St-Louis, MO, USA). For single-cell RT-PCR analysis, individual neurons were visualized by using a phase-contrast microscope and collected into a micropipette. PCR products were electrophoresed on a 2% agarose gel, and were visualized by ethidium bromide staining. Primer sequences used in this study: ADR1 (forward primer) 5' TCC TGA CTG GCT GAA AGA CAA CGA 3', (reverse primer) 5' ACA GTG TGG AAG AGC CAG GAG AAA 3', ADR2 (forward primer) 5'-TGT GCT ACC GGA TTG GCT TAA GGA-3', (reverse primer) 5'-TAC ACC GTG TGG AAG AGC CAT GAA-3', Actin (forward primer) 5'GGC TGT GTC CCAT GTA T 3', (reverse primer) 5'CCG CTC ATT GCC GAT AGT G 3'.

### Immunoblot analysis

Lysates of cultured cells were obtained by washing the cells in ice-cold PBS and resuspending them in cell lysis buffer. Proteins were extracted from ipsilateral mouse brain tissue specimens and 40 μg of protein was separated by SDS-PAGE (8-12%) and then transferred to a nitrocellulose membrane. The membrane was blocked in 5% non-fat milk for 1 h at room temperature, followed by an overnight incubation at 4°C with primary antibodies against: Actin (Sigma); ADR1 (Santacruz, USA; Alexis, USA), ADR2 (Alexis, USA), APPL-1, p-AMPK and Cleaved Caspase-3 (Cell Signaling). Membranes were then washed and incubated with secondary antibodies for 1 h at room temperature °C. Protein bands were visualized using a chemiluminescence detection kit (Amersham Biosciences, Piscataway, NJ, USA).

### Immunocytochemistry and Immunohistochemistry

Neurons grown on 24-chamber microscope slides were fixed in 4% paraformaldehyde and incubated at 4°C with primary ADR1 (Santacruz, USA; Alexis, USA), ADR2 (Alexis, USA) antibodies overnight, followed by a 2 h incubation with FITC conjugated secondary antibody at room temperature. Frozen brain sections were incubated with primary antibodies against ADR1, ADR2 and NeuN (Millipore, Billerica, MA). Images of cells were acquired using a Zeiss Axiophot microscope (Oberkochen, Germany). Formalin-fixed, paraffin embedded human brain sections were incubated with primary antibodies against Adiponectin (Abcam, USA), and biotinylated secondary antibody and avidin-biotin peroxidase complex (Vector Elite Kit; Vector, Burlingame, CA, USA). The peroxidase reaction was developed using a peroxidase substrate kit (diaminobenzidine DAB, SK-4100; Vector).

### Glucose and oxygen deprivation and Cell viability experiments

For glucose deprivation (GD), cultured neurons were incubated in glucose-free Locke's medium containing (in mmol/L) 154 NaCl, 5.6 KCl, 2.3 CaCl_2_, 1 MgCl_2_, 3.6 NaHCO_3_, 5 HEPES, pH 7.2, supplemented with gentamycin (5 mg/L) for 6, 12 or 24 h. For combined oxygen and glucose deprivation (OGD), neurons were incubated in glucose-free Locke's medium in an oxygen-free chamber with 95% N2/5% CO_2 _atmosphere for either 3, 6, 9, 12 or 24 h. Effects of globular adiponectin (gAd) and trimer adiponectin (tAd) (CYT-432, CYT-247 Prospec Bio, Israel) against GD or OGD-induced neuronal cell death were determined by trypan blue dye exclusion assay.

### Statistical Analyses

Statistical comparisons were made by using ANOVA, and Newman-Keuls post hoc tests for pairwise comparisons.

## Results

### ADR1 and ADR2 are expressed in cortical neurons, and their levels increase in response to oxygen and glucose deprivation

**Figure 1:**
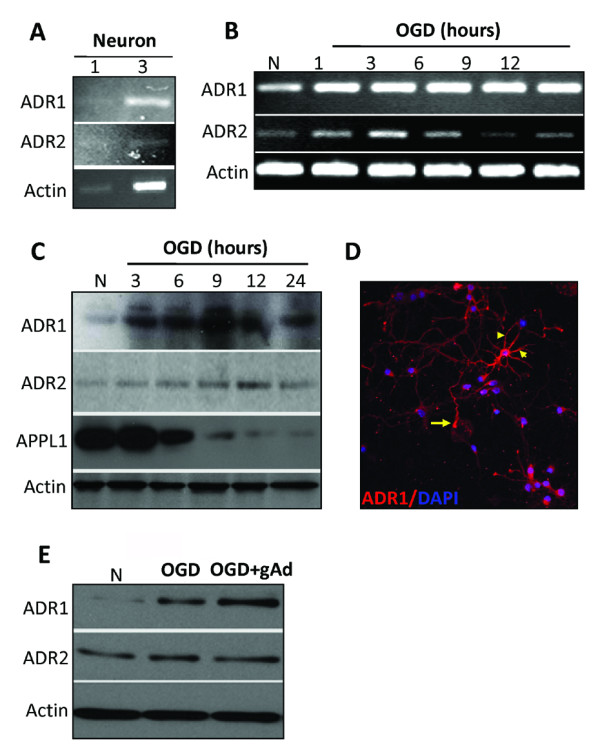
**Neurons express ADR1 and ADR2 and respond to oxygen and glucose deprivation**. **(A) **ADR1 and ADR2 mRNA are present in cultured cortical neurons determined by single-cell RT-PCR analysis. The numbers indicate the number of neurons from which RNA was amplified; 1 and 3 cortical neurons consistently yielded a positive PCR signal for the ADR1 and ADR2 with exactly predicted size. **(B) **Cortical neurons subjected to OGD for the indicated times show increased levels of ADR1 and ADR2 mRNA in a time-dependent manner. **(C) **Immunoblot analysis of proteins in cell lysates of neurons in control cultures and cultures subjected to OGD for 3-24 h. OGD resulted in increased levels of ADR1 and ADR2 **(D) **ADR1 immunoreactivity (red) in cultured neurons; cells were counterstained with DAPI (blue) to label all nuclei. Arrow points to the axon of a neuron and arrowheads point to dendrites of the same neuron. **(E) **Cortical neurons subjected to OGD following globular adiponectin treatment show increased levels of ADR1.

Using single-cell PCR analysis we found that cultured murine cortical neurons express mRNA for ADR1 and ADR2 (Figure 1A). In order to determine whether ADR signaling is involved in neuronal responses to ischemic conditions, we first evaluated ADR expression levels in cultured neurons subjected to OGD. OGD exposure resulted in an increase in the mRNA levels of ADR1, as well as a transient increase in the mRNA levels of ADR2 that peaked at 3 hours (Figure 1B). ADR1 protein levels were also increased during OGD while ADR2 showed a transient increase in protein expression that peaked at 12 hours after the onset of OGD (Figure 1C). Confocal microscopy of ADR1 immunoreactivity showed that ADR1 is present in the cell body, axon and dendrites, but is absent from the nucleus, of cultured cortical neurons (Figure 1D). Addition of globular adiponectin to cultured neurons increased the expression of ADR1 at 6 and 12 hours post-OGD exposure, but had no effect on ADR2 expression levels (Figure 1E).

### Cerebral ischemia induces a rapid increase in ADR immunoreactivity in the brain

**Figure 2:**
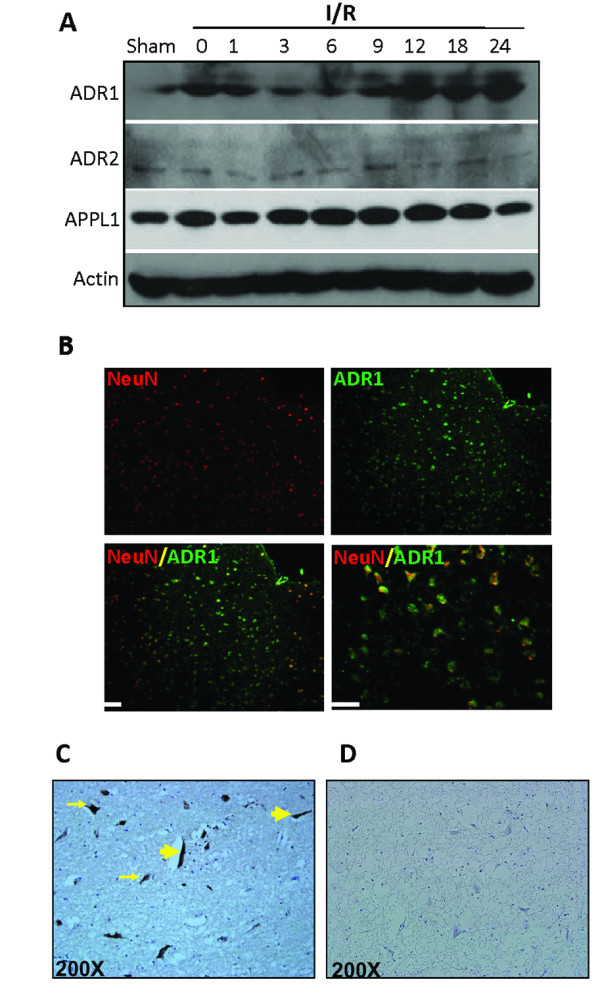
**Cerebral ischemia increases ADRs immunoreactivity in the brain**. **(A) **Immunoblot analysis of protein samples from the cerebral cortex of sham operated control mice and mice subjected to 1 h cerebral ischemia and 1-24 h reperfusion. Ischemia resulted in rapid increases in the levels of ADRs immunoreactivities in neurons in the penumbra area (P). (Scale bars: 50 μM). **(B) **Images of brain sections showing ADR1 immunoreactivities (green) and NeuN (neuronal marker) in mice subjected to cerebral ischemia (1 h) and reperfusion (24 h). **(C) **Adiponectin accumulates in vessels like structures (large yellow arrow) and in parenchyma (small yellow arrow) in the human ischemic brain. **(D) **Control brain tissue stained with secondary antibody shows no staining.

The extensive increase in expression of ADR1 and ADR2 following OGD *in vitro *encouraged us to examine whether similar effects occur *in vivo *following ischemic damage. Immunoblots of ischemic cortical tissues at different times following ischemic reperfusion demonstrated increased levels of ADR1 (Figure 2A). In contrast, the levels of ADR2 remained unchanged following MCAO and reperfusion (Figure 2A). APPL1 is an adapter protein involved in ADR1 and ADR2 signaling and enhances the binding affinities of the ADRs to adiponectin [15]. APPL1 levels were highly expressed in the cortex, and we observed an increase in its expression levels in response to MCAO and reperfusion (Figure 2A). In the ipsilateral cerebral cortex of sham-operated control mice, little or no immunoreactivity with ADR1 and ADR2 antibodies was observed. At 6 h after stroke, neurons in the ischemic cortex exhibited robust ADR1 immunoreactivity (Figure 2B). In order to see adiponectin accumulate in the human brain following ischemic stroke, we analysed brain tissue obtained from a stroke patient at National Taiwan University Hospital. We observed accumulation of adiponectin in vessel-like structres (Large arrows) as well as in parenchyma (small arrows) in human ischemic brainstem (2C).

### ADR activation enhances cortical neuronal death induced by oxygen and glucose deprivation

**Figure 3:**
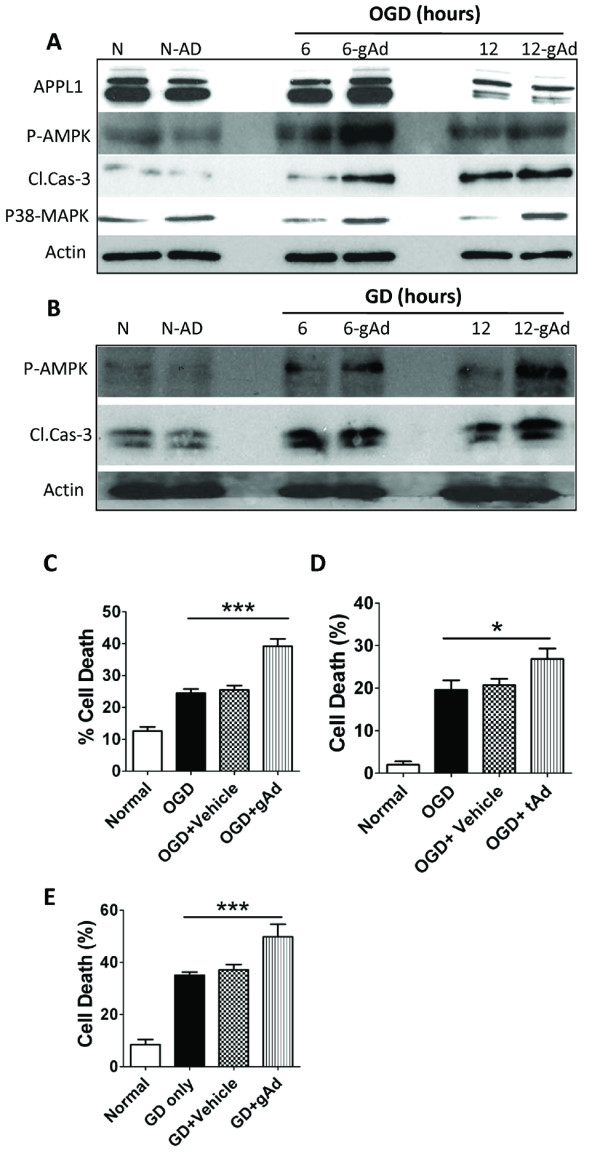
**ADR activation mediates neuronal cell death following *in vitro *ischemia-like conditions**. **(A, B) **Neuronal cultures were treated with 10 μg of the globular adiponectin (gAd) and then subjected to OGD for 12 h **(A) **or GD for 24 h (**B**). Proteins in cell lysates were then subjected to immunoblot analysis by using the indicated antibodies. The gAd treatment enhanced OGD or GD-induced increases in levels of p-AMPK, p-38 MAPK and activated caspase-3. **(C-D) **Globular adiponectin (gAd) and trimeric adiponectin (tAd) treatment exacerbates OGD induced death of cultured primary neurons. Neuronal cell death was quantified 12 h later. Values are mean ± s.e.m. (n = 6-10 cultures). ***P < 0.0001 compared to OGD or vehicle treated OGD value, *P < 0.05 compared to OGD or vehicle treated OGD value. **(E) **Globular adiponectin (gAd) treatment exacerbates GD induced death of cultured primary neurons. Neuronal cell death was quantified 24 h later. Values are mean ± s.e.m. (n = 6-10 cultures). ***P < 0.0001 compared to GD or vehicle treated GD value.

Because the activation of AMPK, p38-MAPK and caspase-3 cleavage and their consequent mitochondrial alterations are implicated in ischemic neuronal death [10,11], and because ADR signaling activates AMPK and p38-MAPK, we next measured levels of cleaved caspase-3, phosphorylated-AMPK (p-AMPK) and p38-MAPK following GD and OGD in globular adiponectin-treated neurons compared with vehicle-treated neurons. Globular adiponectin treatment significantly increased p-AMPK, p38-MAPK and activated caspase-3 levels as compared to vehicle-treated neurons suggesting a pro-apoptotic role for ADRs in neurons under ischemia-like conditions (Figure 3A &3B respectively). Furthermore, we analysed GD- and OGD-induced cell death in adiponectin-treated neurons (10 μg/ml; Prospec, Israel) and compared it with vehicle-treated neurons. Our data showed that both globular and trimeric adiponectin significantly increased cell death in neurons subjected to GD and OGD (Figure 3C-F).

## Discussion

In the brain, so far, adiponectin receptor expression has only been shown in the arcuate and the paraventricular nuclei of hypothalamus [8,9]. We have now identified the presence of both adiponectin receptors (ADRs 1 & 2) in mice cortical neurons. Confocal microscopy of ADR1 immunoreactivity shows that ADR1 is present in the cell body, axon and dendrites, but is absent from the nucleus, of cultured cortical neurons. As seen in the immunoblots of cortical neuron cultures, ADR1 expression was also more prominent *in vivo *following reperfusion, as compared to ADR2. Adiponectin is widely known to promote anti-inflammatory effects such as inhibition of NF-κB, TNF-α, IL-6 and IFN-γ, while increasing levels of IL-10 and IL1RA [16]. These effects are believed to confer protection against chronic disease conditions like atherosclerosis [17] and the metabolic syndrome [18]. To some extent, these protective effects are due to the ability of adiponectin to phosphorylate AMPK via ADRs [19,20]. Conversely, studies investigating the role of AMPK in neuronal survival/death have generated much controversy. The discrepancies, seen in studies performed *in vitro*, are most likely because of the differences in models, culture conditions [21], and the cell type used (e.g., transformed neural tumor cells versus primary cells). Recently, one group used a more direct approach by examining animals with selective gene deletion of AMPKα [10]. AMPKα knockout mice were protected from experimental ischemic stroke compared with wild-type controls. The beneficial effect of the AMPK inhibitor, Compound C, supported this detrimental effect of AMPK after stroke [10]. The combined results from these genetic and pharmacological approaches strongly suggest that activation of AMPK pathways in the brain is detrimental to neuronal survival following ischemia. Since ADR signaling is known to activate AMPK pathways, we hypothesized that the cerebral accumulation of adiponectin and its consequent ADRs activation following ischemic stroke could contribute to neuronal cell death. We therefore, examined the effect of adiponectin treatment *in vitro *on levels of cleaved caspase-3, a hallmark indicator of apoptosis, and phospho-AMPK, in cultured cortical neurons subjected to OGD. Consistent with our hypothesis, we found that ADR1 activation following adiponectin treatment during OGD, enhanced OGD-induced AMPK and caspase-3 activation, as well as neuronal death, thereby suggesting a pro-apoptotic role of ADR 1 activation by adiponectin contributes to neuronal death under ischemic conditions.

The stress-activated p38-MAPK plays important roles in transducing stress-related signals by phosphorylating intracellular enzymes, transcription factors and cytosolic proteins involved in apoptosis and inflammatory cytokine production. Sustained activation of p38-MAPK has been shown to be associated with neuronal cell death/apoptosis following ischemic stroke [22], and inhibition of this pathway is neuroprotective [23]. Our findings suggest that ADR-mediated p38-MAPK contributes to neuronal death after cerebral ischemia by promoting apoptotic cascades in neurons. Caspase-3 mediated apoptosis facilitates synaptic and neurite degeneration early in the ischemic neuronal death process [24], suggesting a role for this mechanism in the pathogenic actions of ADR signaling.

It has been proposed that the globular fragment of adiponectin is generated by proteolytic cleavage, and recently it has been shown that the cleavage of adiponectin by leukocyte elastase, secreted from activated monocytes and/or neutrophils, could be responsible for the generation of the globular fragment of adiponectin [25]. It has been shown previously that adiponectin exerts a cerebroprotective action through an endothelial nitric oxide synthase-dependent mechanism [26]. Nishimura and colleagues showed that Adiponectin-KO mice exhibited enlarged brain infarction and increased neurological deficits after ischemia-reperfusion compared with WT mice [26]. Conversely, systemic administration of adenoviral vectors expressing full-length murine adiponectin significantly reduced cerebral infarct size in WT and Adiponectin-KO mice. However, this murine adiponectin does not exclusively comprise the globular fraction and may have the ability to oligomerise into high, medium and low molecular weight oligomers, thereby inducing ADR2 activation. However, this study has not analysed the role of globular adiponectin which has a higher binding affinity towards ADR1.

The pathophysiological processes in stroke are complex and also involve disruption of the blood-brain barrier (BBB) [27]. It is noteworthy that in response to ischemic stroke, the microvasculature assumes an inflammatory phenotype characterized by leukocyte-endothelial cell adhesion, leukocyte capillary plugging, endothelial barrier dysfunction and activation of resident leukocytes including neutrophils [28]. Although the CNS is normally isolated from the immune system by the BBB, activated leukocytes can easily infiltrate the CNS once the BBB is disrupted during ischemic stroke. This disruption could also facilitate the penetration of full length adiponectin into injured brain tissues, that could be further cleaved by leukocytes elastases at the site of injury [25]. The pathophysiological importance of adiponectin cleavage by leukocyte elastase *in vivo *remains unclear. However, various studies using different cell types have reported a pro-inflammatory role for globular adiponectin [29-31]. These studies showed globular adiponectin to be a potent stimulator of NFκB and other pro-inflammatory genes, which could be detrimental during an inflammatory pathology like stroke. Another recently published study by Bråkenhielm and colleagues showed that adiponectin induced caspase mediated cell death in endothelial cells [32]. Notably, the physiological levels of adiponectin in both human and mouse serum have been reported to range from 2 to 17 μg/ml and also elevated following inflammatory disease conditions like preeclampsia and arthritis [29,33,34]. Thus, the concentration of adiponectin used in our study (10 μg/ml) is well within the observed physiological concentration.

## Conclusions

This study reveals a novel pathogenic role for adiponectin and adiponectin receptor activation in ischemic stroke. We show that cortical neurons express adiponectin receptors and levels of ADR1 increase substantially under ischemic conditions, and that addition of globular or trimeric adiponectin to neurons exacerbates cell death. Our results suggest that ischemia-induced neuronal ADR1 expression may increase the sensitivity of neurons to circulating levels of adiponectin following stroke, contributing to disease pathogenesis.

## List of abbreviations

**ADRS**: Adiponectin receptors; **AMP**: Adenosine monophosphate; **AMPK**: AMP-activated protein kinase; **APPL-1**: Adaptor protein, phosphotyrosine interaction, PH domain and leucine zipper containing 1; **BBB**: Blood Brain Barrier; **CNS**: Central nervous system; **CCA**: Common carotid artery; **ECA**: External carotid artery; **GAD**: Globular adiponectin; **GD**: Glucose deprivation; **ICA**: Internal carotid artery; **MAPK**: Mitogen-activated protein kinase; **MCA**: Middle cerebral artery; **MCAO**: Middle cerebral artery occlusion; **NFκB**: Nuclear factor-kappa B; **OGD**: Oxygen and glucose deprivation; **P38-MAPK**: p38 mitogen-activated protein kinase; **RT-PCR**: Reverse transcription polymerase chain reaction; **SDS-PAGE**: Sodium dodecyl sulfate polyacrylamide gel electrophoresis; **TAD**: Trimeric adiponectin.

## Competing interests

The authors declare that they have no competing interests.

## Authors' contributions

JT designed the study, performed animal and cell culture experiments and analyzed data. ET and KS performed cell culture experiments and analyzed data. SCT and YIL collected human tissue and performed immunohistochemistry experiments. VTK, TMW, SMT, DGJ and MPM provided lab facilities, helped to interpret data and wrote the manuscript. TVM designed the study, provided lab facilities, helped to interpret data and wrote the manuscript. All authors read and approved the final manuscript.
